# Deep gray matter atrophy mediates the associations between glymphatic dysfunction and clinical disability in relapsing-remitting multiple sclerosis: a neuroimaging subgroup study

**DOI:** 10.3389/fimmu.2026.1758859

**Published:** 2026-03-03

**Authors:** Hao Zhang, Ruisi Gong, Hefei Fu, Jinlin Jiao, Mengyao Li, Zixuan Song, Ziying Zhang, Yueluan Jiang, Ye Han, Feng Shi, Jibin Cao, Lingling Cui

**Affiliations:** 1 Department of Radiology, The First Hospital of China Medical University, Shenyang, Liaoning, China; 2 Department of Neurology, The First Hospital of China Medical University, Shenyang, Liaoning, China; 3 MR Research Collaboration Team, Siemens Healthineers China, Beijing, China; 4 Department of Research and Development, United Imaging Intelligence, Shanghai, China

**Keywords:** deep gray matter volume, DTI along the perivascular space, DTI-ALPS, glymphatic function, multiple sclerosis

## Abstract

**Background:**

Impaired glymphatic function is linked to cerebral atrophy and contributes to clinical disability in patients with relapsing-remitting multiple sclerosis (RRMS). Deep gray matter volume (DGMV) loss is associated with disability; however, its mediating effect in MS-related disability and glymphatic function changes remains underexplored.

**Methods:**

One hundred and thirty-one RRMS patients and 50 healthy controls (HC) underwent MRI scans. The DTI-ALPS index was used to evaluate glymphatic function. Z-scores of cortical and deep gray matter volumes (CGMV and DGMV) and WM-FA in RRMS patients were determined based on the mean and standard deviation of HC. RRMS patients were divided into two subgroups: the “MS-DGM-preserved” subgroup (z-scores of both CGMV, DGMV, and WM-FA > -2) and the “MS-DGM-atrophied” subgroup (z-scores of DGMV < -2) according to combinations of z-scores compared to HC. The mediating effect of DGMV in the relationship between the DTI-ALPS index and the clinical disability was further explored. Patients were followed up and had longitudinal outcomes.

**Results:**

Among all participants, 79 cases (60.3%) were classified as the MS-DGM-preserved subgroup, and 52 cases (39.7%) as the MS-DGM-atrophied subgroup. The MS-DGM-atrophied subgroup exhibited lower DTI-ALPS index (d=1.42, p-FDR< 0.001), higher T2-hyperintense white matter lesion volume (d=0.98, p-FDR< 0.001) and EDSS scores (d=0.49, p-FDR< 0.001), and longer disease duration (d=0.33, p-FDR=0.005) compared to the MS-DGM-preserved subgroup. Additionally, in the MS-DGM-atrophied subgroup, the DTI-ALPS index was significantly positively correlated with DGMV (r=0.59, p-FDR<0.001), and negatively correlated with EDSS scores and disease duration (r=-0.59, r=-0.56, p-FDR<0.001). Mediation analysis revealed that DGMV partially mediated the relationship between the DTI-ALPS index and clinical disability (EDSS and disease duration). In the longitudinal cohort, 18 MS patients were followed for a median time of 14 months (12.75, 14.00 months; range: 8–18 months). Compared to baseline, the DTI-ALPS index significantly decreased during follow-up (d=0.92, p-FDR=0.009).

**Conclusion:**

The RRMS subgroups based on the gradient classification of DGMV using structural MRI effectively distinguishes differences in glymphatic function and clinical disability. When DGM atrophy reaches a certain threshold, it partially mediates the relationship between glymphatic function and clinical disability.

## Introduction

Multiple sclerosis (MS) is an immune-mediated chronic inflammatory disease of the central nervous system (CNS) and the leading cause of neurological disability in young individuals. Its pathogenesis is characterized by demyelination, axonal loss, and neurodegeneration (1). Neurodegeneration plays a crucial role in disability progression and is reflected *in vivo* by reduced brain volume or brain atrophy (2). As research on the pathogenic mechanisms of MS advances, current therapeutic strategies focus not only on controlling inflammation but also on addressing neurodegeneration. Monitoring brain volume loss provides a more comprehensive assessment of MS patients’ conditions and allows for more accurate predictions of disease progression and deterioration (3). Studies have shown that brain atrophy occurs early in MS, even before clinical symptoms appear, at a rate far exceeding normal aging, particularly deep gray matter (DGM) atrophy, which occurs at all stages of the disease (4–6). The extent of DGM atrophy has a broader impact than atrophy in other regions, with its degree of atrophy associated with physical disability and cognitive impairment (4, 5, 7, 8). It also affects information processing speed (9). A longitudinal study found that significant DGM atrophy was strongly associated with the progression of persistent disability after 5 years, compared to stable disability (10).

In recent years, research on the cerebral glymphatic system has gained significant attention and become a focal point in the field of neuroscience. The glymphatic system is a highly organized fluid clearance pathway and is believed to play an important role in waste removal in various neuroinflammatory and neurodegenerative disorders. In 2017, Taoka et al. developed a noninvasive assay based on diffusion tensor imaging analysis of the perivascular space (DTI-ALPS index) to assess glymphatic function (11). Although the DTI-ALPS index measures the diffusion rate of peripheral white matter at the level of the lateral ventricles in the direction of the perivascular space, it is recognized as an indirect indicator of glymphatic function and has been applied in a variety of diseases, including Alzheimer’s disease (12, 13), gliomas (14, 15), neuromyelitis optica spectrum disorder (16), and Parkinson’s disease (17, 18), among others. Glymphatic dysfunction has been shown to be associated with cerebral atrophy and to impact clinical disability in MS. Previous studies have indicated that DGM atrophy is accompanied by a decline in glymphatic function (19–21). However, its role in mediating MS-related disability and glymphatic function changes remains understudied.

The clinical classification of MS is primarily based on its clinical manifestations and transformations, including clinically isolated syndrome (CIS), primary progressive MS (PPMS), secondary progressive MS (SPMS), and relapsing-remitting MS (RRMS), as well as on disease activity and progression, characterized by phenotypic features such as active or inactive, and worsening or progressive (22–24). However, while this classification provides a standardized and widely accepted framework, it can present challenges due to its reliance on subjective recall of symptoms and interpretation of signs. Previous imaging, immunologic, or pathologic examinations typically reveal more similarities than differences between MS clinical phenotypes (23, 25). MRI is a strong candidate for data-driven, disease-based classification, as it reflects the pathogenic mechanisms of MS more accurately and complement clinical assessments than a purely clinical diagnosis (26, 27). Increasingly, studies are redefining MS subgroups, mainly focusing on the RRMS, based on structural MRI features (including DGMV) that better reflect the pathogenic mechanisms of MS and predict disease progression (28, 29).

**Figure 1:**
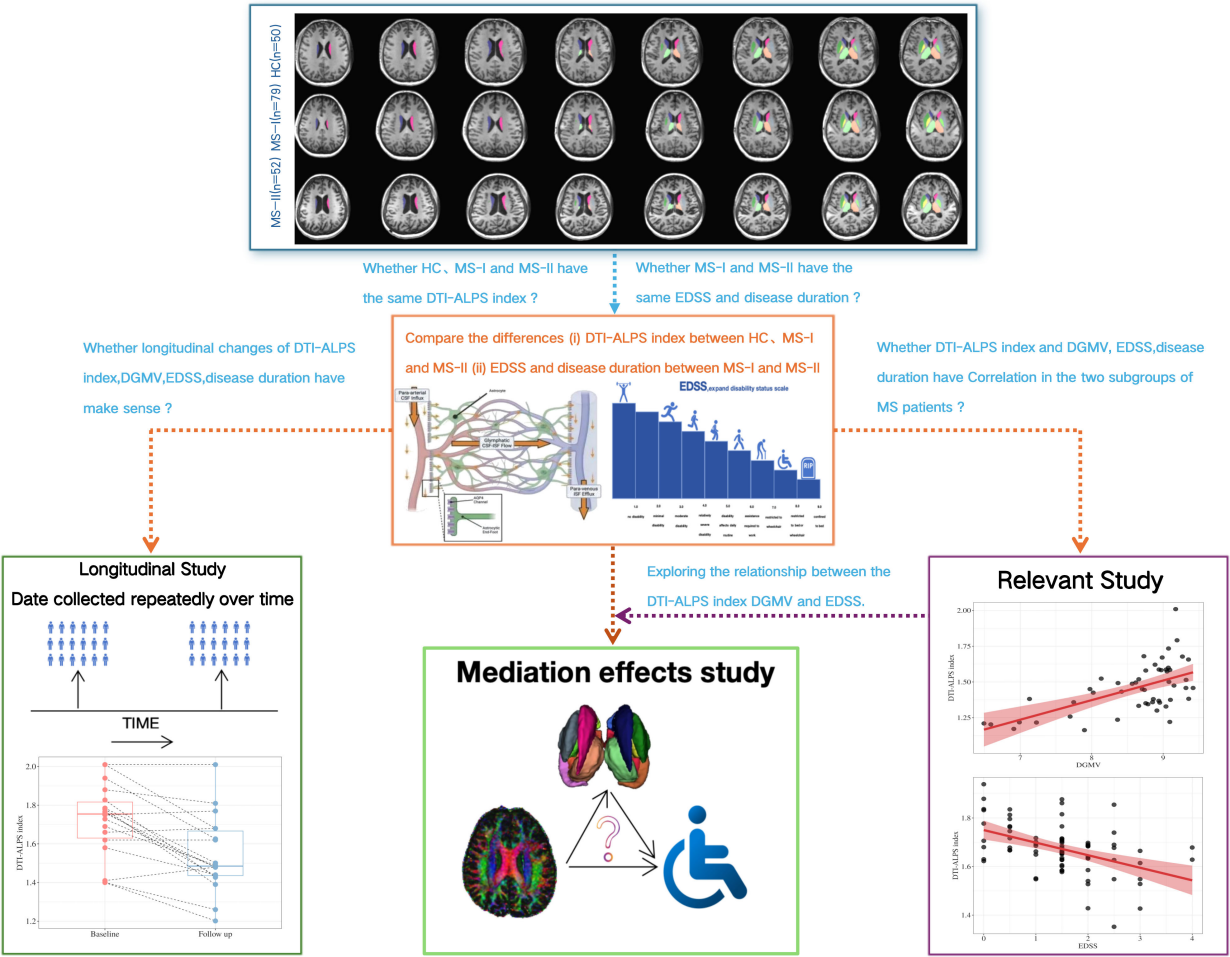
Flow diagram of study design.

Based on this background, the aim of this study is to (i) establish subgroups of RRMS based on the gradient classification of DGMV using structural MRI, (ii) conduct a comparative analysis of the potential relationships among DGMV atrophy, glymphatic function impairment, and clinical disability, and (iii) explore their longitudinal changes (see **Figure 1**).

## Materials and methods

### Participants

**Figure 2:**
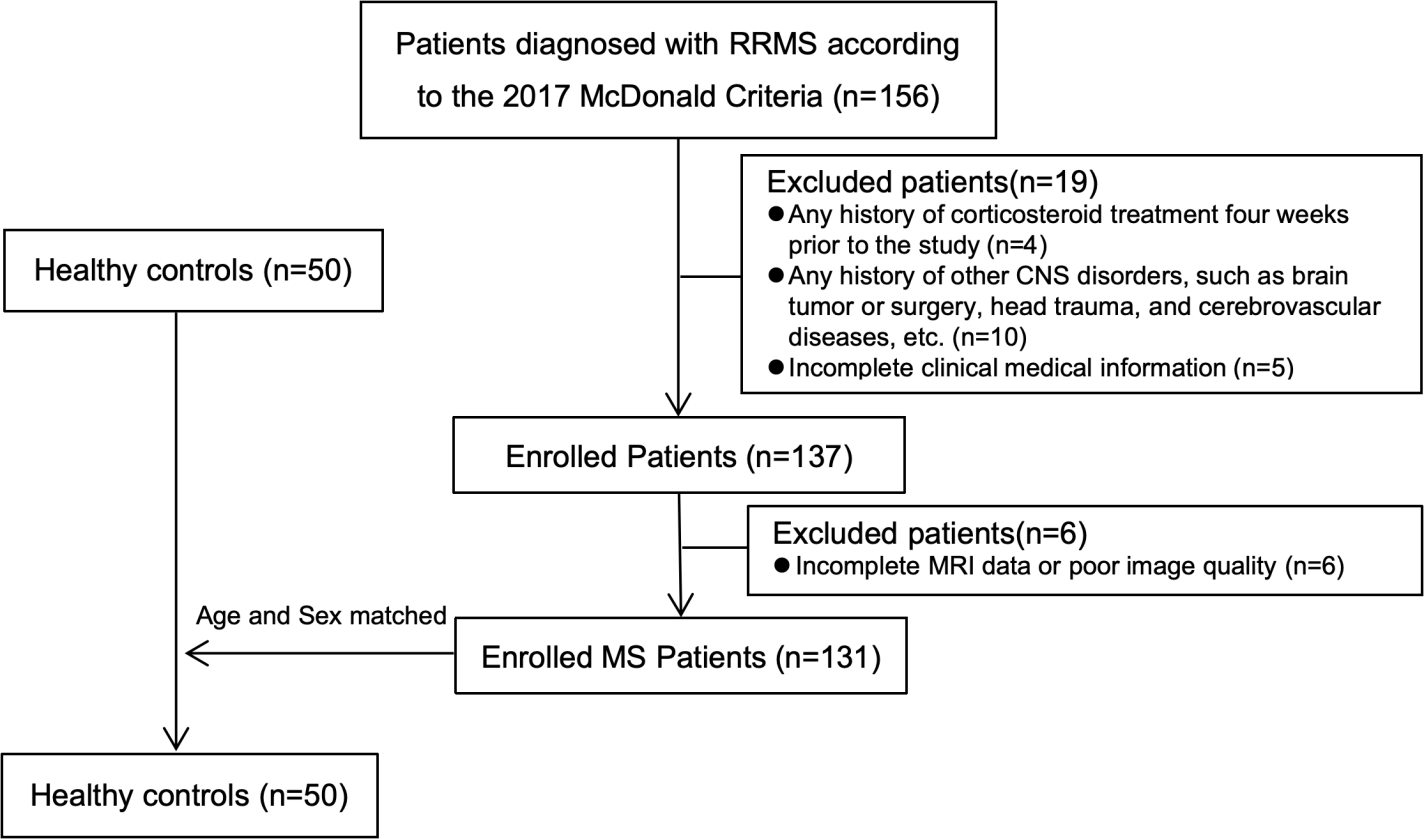
Flowchart for selecting RRMS patients and healthy controls.

The study was approved by the local ethics committee, and informed consent was obtained from all participants. All MS patients were recruited from the Department of Neurology between October 2019 and April 2024. Patients diagnosed with RRMS according to the 2017 McDonald criteria and who had complete MRI scans during a relapse-free state were included. Within 3 days from MRI acquisition, all patients with RRMS underwent a complete neurologic examination, with the Expanded Disability Status Scale (EDSS) score rating and recording of disease-modifying treatments, performed by a neurologist blinded to MRI findings. All participants were right-handed. Fifty age- and sex-matched healthy controls (HC) were also included in the study. Exclusion criteria included: (1) any history of corticosteroid treatment within four weeks prior to the study; (2) any history of other CNS disorders, such as brain tumors or surgeries, head trauma, or cerebrovascular diseases; (3) incomplete clinical information; (4) any MRI contraindications; and (5) incomplete MRI data or poor image quality. (see **Figure 2**). Z-scores of cortical and deep gray matter volumes (CGMV and DGMV) and white matter fractional anisotropy (WM-FA) were calculated for RRMS patients based on the mean and standard deviation of HC (28). We classified RRMS patients into two subgroups: “MS-DGM-preserved subgroup” (z-scores of CGMV, DGMV, and WM-FA > -2) and “MS-DGM-atrophied subgroup” (z-scores of DGMV < -2). In the longitudinal study, 18 RRMS patients participated in the follow-up, with a mean follow-up time of 14 (12.75, 14.00) months (range: 8–18 months). All patients received DMT therapy during the follow-up period and were relapse-free at scanning. In accordance with international clinical guidelines, stable DMT efficacy in MS is defined by sustained abrogation of clinical activity (no confirmed relapses/definitive EDSS progression), absent radiological inflammation (no new gadolinium-enhancing/T2-hyperintense lesions) and attenuated neurodegeneration (no excessive brain atrophy) over continuous follow-up (≥6 months for short-term, ≥24 months for long-term stability), with all assessments excluding non-disease-induced pseudo-relapses.

### Image acquisition

All patients underwent an MRI scan of the brain on a 3.0-T system (SIGNA Pioneer, General Electric, Madison, United States) using a twenty-one-channel phased-array head coil. The imaging protocol comprised a sagittal 3D-T2-fluid-attenuated inversion recovery (3D-T2-FLAIR) with the following parameters: repetition time (TR) = 6800.0 ms, echo time (TE) = 100.0 ms, echo train length (ETL) = 185, matrix dimensions of 240 × 240, field of view (FOV) of 240 × 240 mm², and 172 contiguous 2-mm slices without interslice gap. DTI was conducted using a single-shot spin-echo planar imaging (EPI) sequence with the following specifications: TR/TE = 17000/100.9 ms, matrix dimensions of 120 × 120, FOV of 240 × 240 mm², 65 contiguous 2-mm slices, 25 noncollinear diffusion directions at a b-value of 1000 s/mm², complemented by an axial acquisition without diffusion weighting (b = 0), resulting in a voxel resolution of 2.0 mm³. 3D T1‐weighted imaging was acquired using a 3D fast spoiled gradient-echo sequence, with TR/TE = 7.8/3.0 ms, matrix dimensions of 240 × 240, FOV of 240 × 240 mm², and 176 contiguous 1-mm slices without interslice gap, yielding a voxel size of 1.0 mm³. QSM data were collected with eight echoes, TR/TE = 57.6/5.4 ms, number of excitations (NEX) = 1.00, FOV of 240 × 240 mm², matrix dimensions of 320 × 320, and a flip angle of 20°. All imaging sequences were aligned parallel to the anterior-commissure to posterior-commissure (AC-PC) line and encompassed the entire cerebrum.

### MRI image processing

MRI analysts were blinded to clinical data, including EDSS scores and subgroups classifications, to minimize bias. All MRI scans were processed using the uAI Research Portal image analysis tool (Shanghai United Imaging Intelligence Co. Ltd) (30, 31). Briefly, the preprocessing steps included skull stripping, bias correction, and resampling of the images to a resolution of 1×1×1 mm³. 3D T1-weighted images were segmented into GM, WM, and CSF, and further parcellated into 109 major regions of interest (ROIs) according to the DK atlas (32). CGMV and DGMV, including the bilateral thalamus, caudate, putamen, pallidum, hippocampus, amygdala, accumbens area, and ventral diencephalon, were obtained. The segmentation was performed using a pre-trained cascaded V-Net model, which combines coarse localization and segmentation refinement. This approach has proven useful in medical image segmentation tasks, including brain tumor segmentation (33).

**Figure 3:**
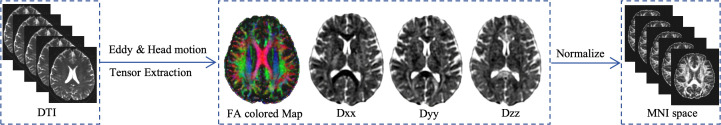
DTI data pre-processing. Images in x, y and z axis are obtained. Nonlinear registration to standard space.

DTI processing was carried out using FMRIB’s Diffusion Toolbox (FDT, http://www.fmrib.ox.ac.uk/fsl). After skull removal and eddy current correction, the FA map and x, y and z-axis diffusion maps were generated using FSL command line “dtifit”. FA map was calculated based on the DTI tensor for each voxel. FA images were normalized to a pre-defined target FA template (FMRIB58_FA) by non-linear registration. Finally, the mean FA value within tracts defined by the JHU-WM tractography atlas (reflecting both WM lesion and the integrity of normal appearing WM) were calculated (see **Figure 3**).

### Quantitative susceptibility image processing

QSM reconstructions were performed using a MATLAB R2013a-based susceptibility imaging software (STISuite, https://people.eecs.berkeley.edu/~chunlei.liu/software.html). The corrected and combined phase images were acquired by weighting the magnitude of the corresponding channel with the vendor-provided combination method and were unwrapped using a Laplacian-phase method. Then, phase-unwrapped images were used to remove the background field using the V-SHARP method. In order to reduce extreme streaking artifacts caused by large veins, susceptibility maps were generated in the process of field-to-susceptibility inversion by using an improved sparse linear equation and least-square algorithm (streaking artifact reduction for QSM, STAR-QSM).

### Calculation of the DTI-ALPS index

**Figure 4:**
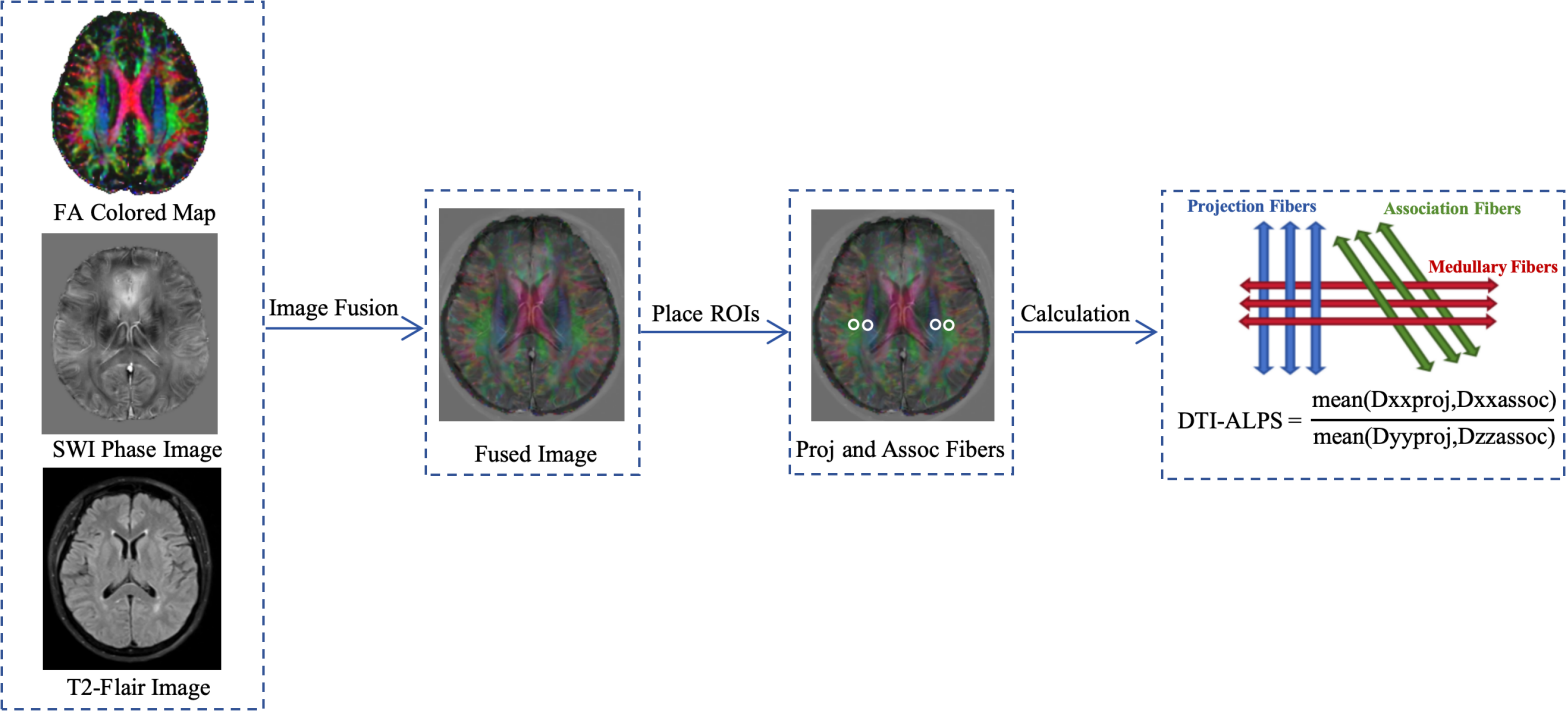
Two regions of interest (ROIs) were drawn on the slice where veins run perpendicular to lateral ventricle on the FA-colored map in quantitative susceptibility mapping (QSM) space. One ROI represented projection fibers (ROI proj) and the other represented associative fibers (ROI assoc) in the both hemisphere. Schematic diagram showed the relationship between the direction of the medullary veins and the direction of the fibers.

With reference to the veins on the QSM, the level at which the medullary vein was located perpendicular to the lateral ventricle was selected for each participant to accurately select the brain region on the x-axis where the space around the vein was located. Two neuroradiologists (5 and 10 years of experience, respectively) placed two regions of interests (ROIs) of 5mm in diameter on color-coded FA maps, with one ROI located in the projection fibers and the other in the association fibers. On T2WI image, each ROI was placed at least 3 mm from the edge of the lesion to avoid the influence of the lesion, and ROIs were drawn in the both hemispheres of the brain to make the relevant fibers sufficiently thick to maximize the likelihood of perpendicularity between the fiber axes and the perivascular gaps, and then the diffusion coefficients along the x-axis, y-axis, and z-axis of each ROI were extracted. The average of the left and right DTI-ALPS index (mean DTI-ALPS index) was calculated as the final DTI-ALPS index. The flowchart is shown below (see **Figure 4**).

### Calculation of the T2-hyperintense white matter lesion volume

First, all 3D-T2-FLAIR images were registered to the MNI152 template using ANTs. Then, brain masks were applied to the images for skull removal. Cropping was performed so that the size of all images was standardized to 160×196×160 with a voxel size of 1×1×1 mm^3^. Segmentation of RRMS T2-hyperintense WM lesions in skull-stripped standard space images was performed using the MoME brain lesion segmentation model (34). In order to eliminate potential effects of interindividual variability in brain size, individual RRMS T2-hyperintense WM lesions was estimated as the ratio of lesions volume to intracranial volume.

### Statistical analysis

Statistical analyses were performed using SPSS statistical software (Version 26.0.0, IBM). A statistical significance threshold of two-sided p<0.05 was adopted. Linear mixed models were employed to account for variability in structural and functional MRI measurements, ensuring robust handling of potential correlations in the data and adjusting for gender, age, the interaction between gender and age, and total intracranial volume (for CGMV and DGMV). The mean residuals from the linear mixed regression models were used as the adjusted MRI measurements in subsequent analyses. For demographic characteristics and clinical variables, chi-square tests, independent samples t-tests, and Mann-Whitney U tests were used for categorical and continuous variables, respectively. ANCOVA, adjusted for age and sex, was performed to compare differences between HC, MS-DGM-preserved subgroup, and MS-DGM-atrophied subgroup. Age- and sex-adjusted correlation analyses were conducted to assess associations between DGMV and the DTI-ALPS index with EDSS and disease duration. Statistically significant variables from the correlation analysis were entered as potential covariates in multivariable linear regression analysis to identify independent factors associated with DGMV and the DTI-ALPS index. Model-based mediation analysis were performed using PROCESS (version 3.5) for SPSS. The “Model 4” function for the mediation model, adjusted for age and sex, was used to explore the potential mediating effect of DGMV between glymphatic function and EDSS and disease duration in MS-DGM-atrophied subgroup patients. We used the nonparametric permutation test to assess the indirect effect in mediation analysis given the small sample size (n=52). Based on 5000 bootstrap resamples, the 95% bias correction confidence interval (CI) of indirect effects was estimated. For the longitudinal analysis, the Wilcoxon signed-rank test was used to compare MRI measurements between baseline and follow-up. Age, sex, and follow-up duration were adjusted to explore the relationships between DGMV, the DTI-ALPS index, and EDSS.

## Results

### Subgroups of RRMS patients and demographic characteristics

The demographic information of the 131 RRMS patients and HCs is displayed in **Table 1**. RRMS patients were classified into subgroups based on the CGMV, DGMV, and WM-FA, which were adjusted for gender, age and education years, the interaction of gender, age, education years, and total gray matter volume (for CGMV and DGMV) by linear mixed models. For all structural MRI measurements, the mean and standard deviation (SD) of the corresponding measurements in HC (CGMV: 495.53 ± 49.98; DGMV: 55.88 ± 5.15, WM-FA: 0.47 ± 0.02) were used to compute z-scores. The z-scores of MRI measurements in RRMS patients were defined as follows: z-score = (MRI measurements in RRMS - mean value of MRI measurement in HC)/SD of MRI measurements in HC. If z-scores of RRMS patients were not below -2 for any of the three measurements, the RRMS patients were designated as the “MS-DGM-preserved” subgroup, which was obtained in 79 cases (60.3%). If z-scores of RRMS patients were below -2 for DGMV, the RRMS patients were designated as the “MS-DGM-atrophied” subgroup, which was obtained in 52 cases (39.7%) (see **Table 2**).

**Table 1:** Demographic information of the HC and RRMS subgroups.

Characteristics	HC(n=50)	MS-DGM-preserved(n=79)	MS-DGM-atrophied(n=52)	*P* value
Gender, female ratio (%)	21(42%)	32(40.5%)	25(42.3%)	0.683^a^
Age(years)	32.18±11.75	34.30±11.52	34.90±9.51	0.433^b^
Education(years)	14.78±2.50	14.17±2.80	14.61±2.90	0.481^b^
DMT status(%):first line/second line	/	34(43.1%)/45(56.9%)	23(44.2%)/29(55.8%)	0.893^a^
DMT status duration(years)	/	2.75±3.42	4.93±4.65	**0.007^c^**
Disease duration(years)	/	2.79±3.45	5.00±4.67	**0.005^c^**
EDSS scores	/	1.47±0.92	2.15±0.98	**<0.001^c^**
T2-hyperintense WM LV(ml)	/	4.28±4.03	11.13±10.00	**<0.001^c^**

The data were shown as the mean values±standard deviation. EDSS, expanded disability status scale; HC, healthy controls; DMT, disease modifying treatment; T2-hyperintense WM LV, T2-hyperintense white matter lesion volume. ^a^P value was obtained using the chi-square test; ^b^P value was obtained using the independent samples t-test; ^c^P value was obtained using the non-parametric test. First-line DMT: interferon β-1a, dimethyl fumarate, teriflunomide, glatiramer acetate; 2nd line DMT: natalizumab, fingolimod, siponimod, ocrelizumab, methotrexate. DMT status duration: DMT treatment duration before the current MRI acquisition.

Bold values indicate statistical significance (p < 0.05).

**Table 2:** Clinical variables of the HC and RRMS subgrous.

Characteristics	HC (n=50)	MS-DGM-preserved(n=79)	MS-DGM-atrophied(n=52)
TIV (ml)	1449.27±126.51	1351.46±112.91^a^	1390.42±100.56^b^
CGMV(ml)	495.53±49.98	465.81±40.48^a^	436.11±40.58^b^
DGMV(ml)	55.88±5.15	49.56±4.60^a^	42.02±5.76^b^
WM-FA	0.47±0.02	0.42±0.04^a^	0.38±0.05^b^

The data were shown as the mean values±standard deviation. HC, healthy controls; TIV, total intracranial volume; CGMV, cortical gray matter volume; DGMV, deep gray matter volume; WM-FA, whiter matter fractional anisotropy. The continuous data and ranked data were analyzed using Mann-Whitney U or Kruskal-Wallis test followed by post-hoc multi-comparison with Tukey-Kramer tests. A statistical significance with two-sided p<0.05 was adopted. ^a^ indicated p<0.05 compared to HC; ^b^ indicated p<0.05 compared to MS-DGM-preserved.

### Comparison between the two subgroups of RRMS patients and HC group

**Figure 5:**
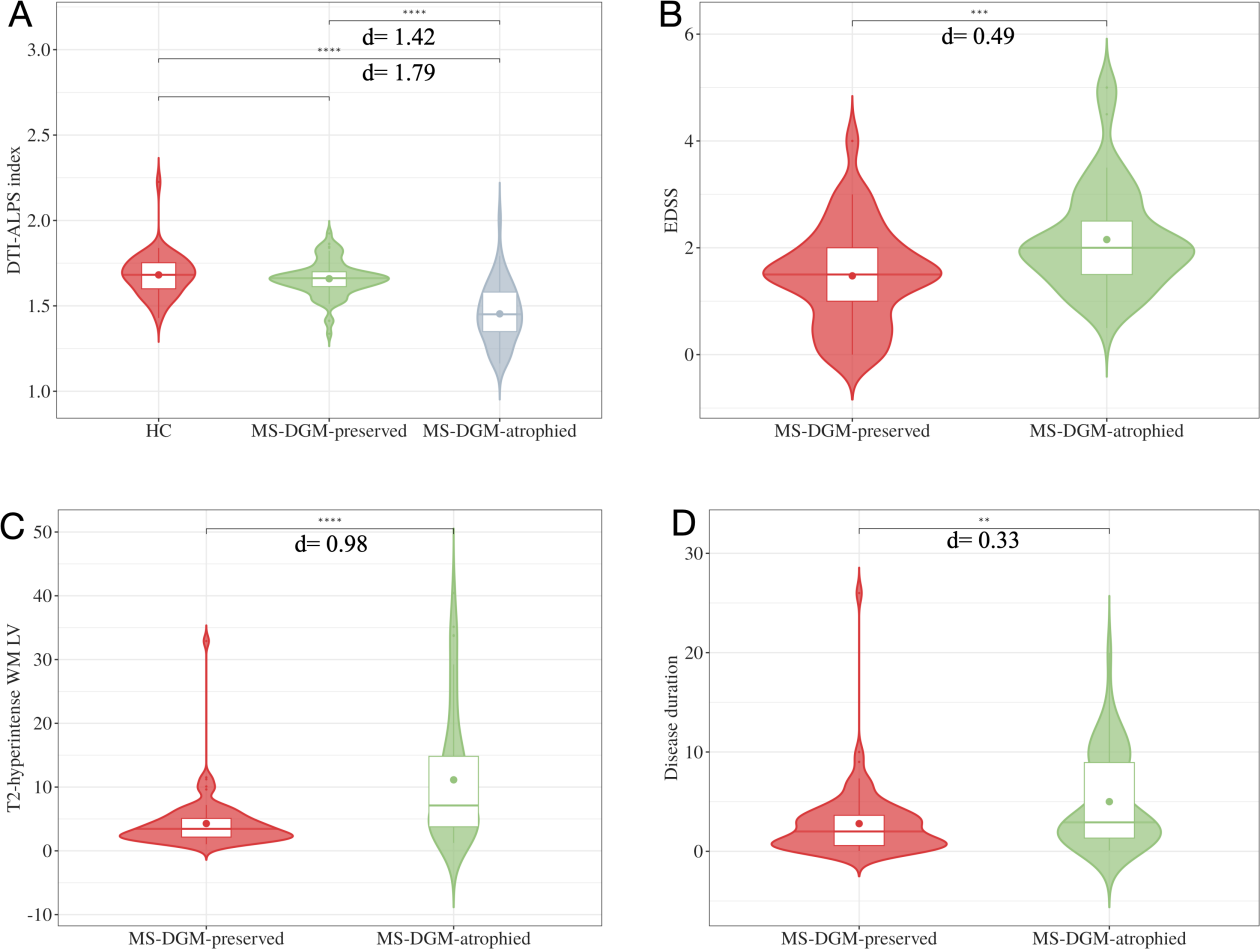
Comparison between the two subgroups of RRMS patients and HC group. The differences in DTI-ALPS index **(A)**, EDSS **(B)**, T2-hyperintense WM LV **(C)** and disease duration **(D)** between the MS-DGM-preserved subgroup and MS-DGM-atrophied subgroup were statistically significant.

There was no difference in the DTI-ALPS index between HC and MS-DGM-preserved subgroup (d=0.16, p-FDR=0.26). Compared with HC, the difference in the DTI-ALPS index in the MS-DGM-atrophied subgroup was statistically significant (d=1.79, p-FDR<0.0001). The differences in DTI-ALPS index (d=1.42, p-FDR<0.001), EDSS (d=0.49, p-FDR<0.001), T2-hyperintense WM lesions volume (d=0.98, p-FDR<0.001), and disease duration (d=0.33, p-FDR=0.005) between the two subgroups were statistically significant, with a significantly lower DTI-ALPS index, a higher EDSS, a higher T2-hyperintense WM lesions volume, and a longer disease duration in MS-DGM-atrophied subgroup (see **Figure 5**).

### Correlation analysis in the two subgroups of RRMS patients

**Figure 6:**
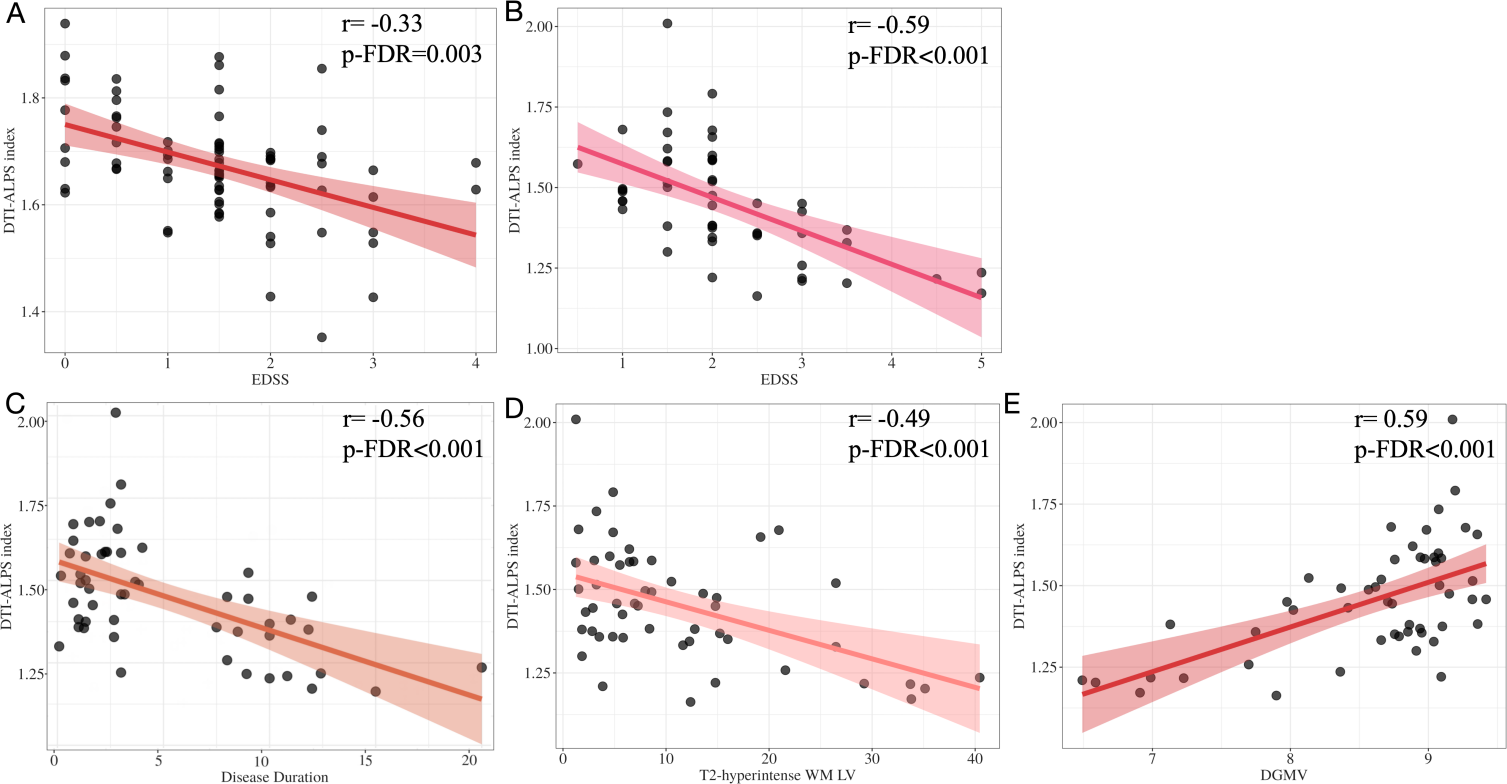
Correlation analysis in the two subgroups of RRMS patients. The DTI-ALPS index showed a negative correlation with EDSS in MS-DGM-preserved subgroup **(A)**. In the MS-DGM-atrophied subgroup, the DTI-ALPS index showed a significant negative correlation with EDSS **(B)**, disease duration **(C)**, T2-hyperintense WM LV **(D)** and significantly positive correlation with DGMV **(E)**.

After adjusting for age, sex and education years, there was no significant correlation between DGMV and EDSS in the MS-DGM-preserved subgroup (p-FDR=0.34), while the DTI-ALPS index showed a negative correlation with EDSS (r=-0.33, p-FDR=0.003). In the MS-DGM-atrophied subgroup, the DTI-ALPS index showed a significant positive correlation with DGMV (r=0.59, p-FDR<0.001) and a significant negative correlation with EDSS, disease duration and T2-hyperintense WM LV (r=-0.59, r=-0.56, r=-0.49, all p-FDR<0.001) (see **Figure 6**). A significant but modest correlation was observed between WM-FA and DTI-ALPS index (r=0.29, p-FDR=0.039), and a similar modest negative correlation with EDSS (r=-0.28, p-FDR=0.046).

### Mediation analysis of DGMV, DTI-ALPS index and EDSS, disease duration

**Figure 7:**
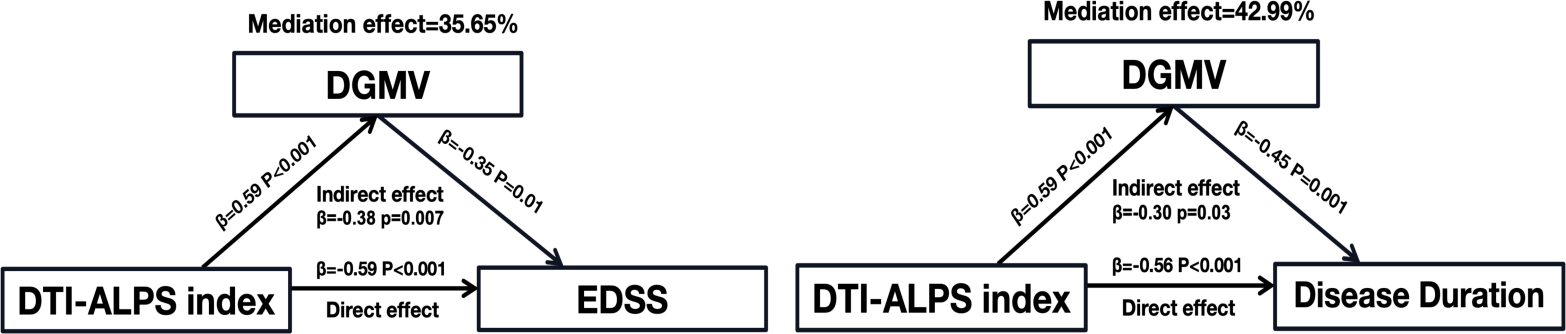
The DGMV partially mediated the association between the DTI-ALPS index and EDSS, disease duration. β, standardized coefficient.

To investigate the effects of DGMV and DTI-ALPS index on clinical disability and disease duration in the MS-DGM-atrophied subgroup, we used the DTI-ALPS index as the independent variable, EDSS and disease duration as the dependent variable, and DGMV as the mediator variable. The analysis revealed that DGMV partially mediated the association between DTI-ALPS index and EDSS (mediation effect = 35.65%, p=0.007), disease duration (mediation effect = 42.99%, p=0.03) in the MS-DGM-atrophied subgroup(see **Figure 7**).

### Longitudinal analysis

**Figure 8:**
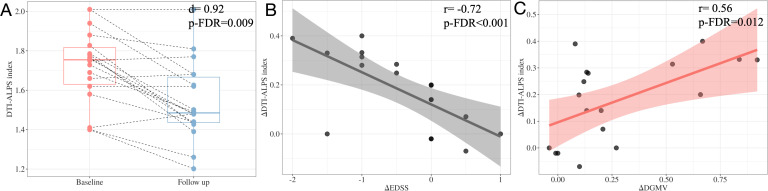
Longitudinal analysis in the DTI-ALPS index between baseline and follow-up **(A)**. Adjusting for sex, age, and follow-up duration, the changes of DTI-ALPS index during follow-up (ΔDTI-ALPS index) showed a significant negative correlation with the changes of EDSS (ΔEDSS) during follow-up **(B)** and a significant positive correlation with the changes of DGMV (ΔDGMV) during follow-up **(C)**.

The general characteristics of the follow-up patients, along with their baseline and follow-up clinical indicators, are shown in **Table 3**. At follow-up, we found that only the DTI-ALPS index was significantly lower compared to baseline (d=0.92, p-FDR=0.009) (see **Figure 8**, **Table 3**), and no significant difference was found in DGMV and EDSS (all p-FDR>0.05).

**Table 3:** Demographic information and clinical variables of the follow-up RRMS patients.

Characteristics	RRMS at baseline(n=18)	RRMS at follow up(n=18)	*P* value
Gender, female ratio (%)	8(44.44%)	8(44.44%)	/
Age(years)	33.72±10.80	35.11±10.76	0.702^b^
Follow-up duration(years)	/	13.31±2.05	/
DMT status(%):first line/second line	8(44.44%)/10(55.56%)	8(44.44%)/10(55.56%)	/
DMT status duration(years)	1.98±1.85	3.08±1.88	0.049^a^
Disease duration(years)	2.05±1.90	3.16±1.93	0.051^a^
EDSS scores	1.53±0.88	1.97±0.90	0.131^a^
T2-hyperintense WM LV(ml)	4.56±3.39	4.63±2.99	0.752^a^

The data were shown as the mean values±standard deviation. EDSS, expanded disability status scale. DMT, disease modifying treatments; T2-hyperintense WM LV, T2-hyperintense white matter lesion volume. ^a^P value was obtained using the non-parametric test. ^b^P value was obtained using the independent samples t-test. First-line DMT: interferon β-1a, dimethyl fumarate, teriflunomide, glatiramer acetate; 2nd line DMT: natalizumab, fingolimod, siponimod, ocrelizumab, methotrexate. DMT status duration: DMT treatment duration before the current MRI acquisition.

Notably, the change in DTI-ALPS index (△DTI-ALPS index) showed a positive correlation with the change in DGMV (△DGMV) (r=0.56, 95%CI[0.11, 0.82], p-FDR=0.012) and a negative correlation with the change in EDSS (△EDSS) (r=-0.72, 95%CI[-0.89, -0.37], p-FDR<0.001) during the follow-up period (see **Figure 8**).

## Discussion

In this study, we established a new subgroup of RRMS based on the gradient classification of DGMV using structural MRI. This stratification serves as an exploratory tool to investigate gradients of DGM atrophy in relation to glymphatic function and disability, rather than proposing new clinical subtypes. Observed differences between the two subgroups likely represent varying degrees of cumulative disease burden rather than entirely novel biological mechanisms. These subgroups effectively enabled the analysis of potential relationships among DGMV atrophy, glymphatic function impairment, and clinical disability. Our results showed no significant impairment of glymphatic function in MS-DGM-preserved subgroup with mild DGM atrophy. However, a higher degree of DGM atrophy (MS-DGM-atrophied subgroup) was more closely associated with glymphatic dysfunction and clinical disability. Moreover, when DGM atrophy reached a certain threshold, it partially mediated the link between glymphatic function and clinical disability.

The pathological events underlying DGM atrophy remain unclear, but it is generally interpreted as a consequence of neurodegeneration (8). MS has been suggested to cause brain structural changes, including cortical thinning and DGM atrophy (19). DGM atrophy has been reported in the early stages of MS and is considered a key predictor of disability (4, 6, 10), Previous studies have demonstrated that patients with progressive MS experience more severe DGM atrophy, longer disease duration, and worse cognitive performance (35). Our research results are consistent with theirs. Histological studies have shown alterations in DGM in MS due to inflammatory and neurodegenerative processes (36, 37). Additionally, studies using multimodal MRI imaging have reported abnormal iron deposition (38, 39), and reduced perfusion (40). Effective immunomodulatory therapy can delay DGM atrophy and disease progression in MS patients (41, 42). Therefore, gradient classification based on DGM atrophy may hold potential value for further investigation of MS mechanisms, though its applicability to hierarchical diagnosis and treatment requires validation in larger cohorts.

We also found a very interesting result: only when DGM atrophy reaches a certain level does its glymphatic function begin to decline, with longer disease duration and higher degrees of disability. Although DGM atrophy plays an important role in the progression of disability in MS, the degree of atrophy itself is also an important factor to consider. DGM structures are extensively connected with cortical GM regions, and therefore, DGM atrophy could result from retrograde and anterograde neurodegeneration through tracts linking GM areas. For example, the extent of cellular density loss in the thalamus is associated with neurodegeneration in remote (but connected) cortical regions, beyond the extent of atrophy explained by demyelination in connecting tracts (37). There is also evidence for other mechanisms of neurodegeneration in the DGM. These structures have a higher iron load than other regions and can accumulate oxidized lipids, which are associated with neurodegeneration and exacerbate clinical disability (43, 44). Moreover, the cerebral glymphatic system, responsible for scavenging through the flow dynamics of cerebrospinal fluid (CSF) and its exchange with interstitial fluid (ISF), plays a critical role in the clearance of CNS wastes. Our results indicate that with the progression of DGM atrophy, the cerebral glymphatic function in MS patients is progressively impaired. This may be due to the accumulation of waste materials, such as iron and oxidized lipids, in the brain, leading to a decline in waste clearance function when DGM atrophy is significant.

Previous findings have shown that glymphatic function is associated with structural brain changes in MS patients (19, 21). We found a closer relationship between glymphatic function impairment and DGM atrophy in the MS-DGM-atrophied subgroup, where DGM was significantly atrophied. Considering the orientation of the glymphatic pathway and the location of regions of interest adjacent to the lateral ventricles, the DTI-ALPS index may better reflect the clearance of toxic molecules in these regions, particularly in the DGM. In addition, mediation analysis revealed that, in the presence of significant DGM atrophy, although decreased cerebral glymphatic function in MS patients was significantly associated with clinical disability, glymphatic dysfunction did not directly lead to disability. Instead, more pronounced DGM atrophy was an important mediator. The effect of glymphatic system damage on clinical disability was mediated by the accumulation of CNS tissue damage, whether due to demyelination or neuronal loss (19). From the early stages of the disease, MS has been reported to cause gray matter damage extending from the lateral ventricles outward (45). Therefore, glymphatic dysfunction may influence gray matter damage due to the failure to remove CSF-derived toxic molecules produced on the DGM surfaces around the ventricles. Similarly, reduced glymphatic fluid flow in the perivenous space may lead to the accumulation of neuroinflammatory triggers in the white matter, contributing to demyelination (46).

In the longitudinal follow-up, we found that only the DTI-ALPS index was significantly reduced and negatively correlated with EDSS scores compared to baseline, while no significant changes were observed in the DGM. These preliminary, hypothesis-generating longitudinal findings, derived from a small sample with variable follow-up durations, necessitate validation in larger prospective studies. DGM atrophy has been reported in the early stages of MS and is recognized as a key predictor of disability (4–8). Our results did not find changes in DGM, possibly due to neurodegenerative changes occurring over a long prodromal period (47). A key obstacle to identifying these disease subtypes may be the insufficiently long follow-up in our longitudinal study. However, in this preliminary cohort, our results showed that the DTI-ALPS index may be a sensitive marker in monitoring the dynamic change of disease progression compared with brain atrophy. Therefore, the DTI-ALPS index shows preliminary promise as a potential marker for monitoring MS progression, though this requires validation in larger, prospective studies. Although the DTI-ALPS index is calculated by extracting diffusion measurements in an artificially defined region of interest, recent studies have shown that this method has good reliability and repeatability (11, 19, 20). Interventions aimed at enhancing brain glymphatic clearance warrant exploration in future research as potential avenues to delay DGM atrophy and disability in MS, pending confirmatory studies. This is consistent with previous studies on the progression of neurodegenerative diseases (48).

Our study has some limitations. First, this is a single-center study and lacks external validation, and thus the results are limited. Further study in a larger sample and a longitudinal design is warranted to validate the current results. Second, the cerebral glymphatic system is a complex structure, and its dispersion along the perivascular space only represents one step in the overall waste removal process. The DTI-ALPS index is a newly developed technology that reflects only the dispersion of the perivascular space around the ventricles and may not fully capture the function of the entire cerebral glymphatic system; its effectiveness remains to be further validated. Third, relapse activity, cognitive assessment, treatment response and follow-up information were incomplete due to the retrospective design of this study. Prospective studies with comprehensive clinical and cognitive assessment are needed and treatment responses of different RRMS subgroups should be investigated. Four, the RRMS subgroups were based on a slightly arbitrary cut-off (2SD) of the z-scores of structural parameters compared to HC; further study is required to identify an optimal method to define the criteria or apply data-driven (e.g. hierarchical and k-mean) methods (49, 50). Lastly, the new subgroups were developed within the RRMS population; generalization to CIS and progressive MS, and those with and without disease activity needs further investigation and may require inclusion of additional MRI parameters (e.g. spinal cord) to better capture the pathological heterogeneity in progressive disease.

## Conclusion

The subgroups of RRMS, based on the gradient classification of DGMV using structural MRI, can effectively distinguish differences in glymphatic function and clinical disability. The greater the degree of DGM atrophy, the more closely it is related to glymphatic function and clinical disability. Additionally, when DGM atrophy reaches a certain level, it partially mediates the link between glymphatic function and clinical disability. Glymphatic function, as evaluated by the DTI-ALPS index, shows preliminary promise as a potential biomarker for monitoring MS progression. In the future, it could potentially inform hierarchical management strategies for RRMS or therapies targeting glymphatic system repair and DGM atrophy delay, but replication in larger, more diverse cohorts is essential before considering any translation to clinical practice.

## Data Availability

The raw data supporting the conclusions of this article will be made available by the authors, without undue reservation.
